# Dual roles of astrocytes in plasticity and reconstruction after traumatic brain injury

**DOI:** 10.1186/s12964-020-00549-2

**Published:** 2020-04-15

**Authors:** Yunxiang Zhou, Anwen Shao, Yihan Yao, Sheng Tu, Yongchuan Deng, Jianmin Zhang

**Affiliations:** 1 Department of Surgical Oncology, The Second Affiliated Hospital, Zhejiang University School of Medicine, No. 88, Jiefang Road, Zhejiang, 310009 Hangzhou China; 2 Department of Neurosurgery, The Second Affiliated Hospital, School of Medicine, Zhejiang University, Province, Zhejiang, 310009 Hangzhou China; 3 State Key Laboratory for Diagnosis and Treatment of Infectious Diseases, Collaborative Innovation Center for Diagnosis and Treatment of Infectious Diseases, The First Affiliated Hospital, College of Medicine, Zhejiang University, Zhejiang, Hangzhou China

**Keywords:** Astrocyte, Traumatic brain injury, Reconstruction, Neurogenesis, Blood-brain barrier, Glial scar

## Abstract

Traumatic brain injury (TBI) is one of the leading causes of fatality and disability worldwide. Despite its high prevalence, effective treatment strategies for TBI are limited. Traumatic brain injury induces structural and functional alterations of astrocytes, the most abundant cell type in the brain. As a way of coping with the trauma, astrocytes respond in diverse mechanisms that result in reactive astrogliosis. Astrocytes are involved in the physiopathologic mechanisms of TBI in an extensive and sophisticated manner. Notably, astrocytes have dual roles in TBI, and some astrocyte-derived factors have double and opposite properties. Thus, the suppression or promotion of reactive astrogliosis does not have a substantial curative effect. In contrast, selective stimulation of the beneficial astrocyte-derived molecules and simultaneous attenuation of the deleterious factors based on the spatiotemporal-environment can provide a promising astrocyte-targeting therapeutic strategy. In the current review, we describe for the first time the specific dual roles of astrocytes in neuronal plasticity and reconstruction, including neurogenesis, synaptogenesis, angiogenesis, repair of the blood-brain barrier, and glial scar formation after TBI. We have also classified astrocyte-derived factors depending on their neuroprotective and neurotoxic roles to design more appropriate targeted therapies.

Video Abstract

Video Abstract

## Background

Traumatic brain injury (TBI) refers to a sudden trauma caused by traffic accidents, wars, violence, terrorism, falls, and sporting activity [1]. TBI is currently the primary cause of human death in young adults and one of the leading causes of fatality and disability across all ages worldwide, resulting in annual global economic losses of amounting to $US400 billion [2–4]. The high mortality and morbidity of TBI and the substantial economic burden affect the patients, families, and society, and have attracted public attention [5]. To date, more than 1000 clinical trials on TBI have been registered on clinicaltrials.gov. In spite of the immense efforts on the treatment of TBI made in the past few decades, few effective therapies for TBI are available [6–8].

One of the reasons for the failure is because most previous studies have targeted neuronal cells, whereas emerging evidence shows that glial cells also play significant roles in the pathogenesis of TBI [9–11]. Astrocytes, a type of glial cells, are involved in the homeostasis and blood flow control of the central nervous system (CNS) [12]. TBI is known to induce astrocyte activation (reactive astrogliosis), which is involved in tissue remodeling processes such as neurogenesis, synaptogenesis, repair of the blood-brain barrier (BBB), regulation of synaptic plasticity, and formation of glial scar and extracellular matrix (ECM), weighing a lot to the patient outcome [13–15]. However, reports on the effects of reactive astrogliosis are not consistent [10, 16–18]. The current review summarizes the existing knowledge on the role of astrocytes in TBI. We particularly elaborate on the various roles of astrocytes and astrocytes-derived molecules in plasticity and reconstruction and explore the possibility of using astrocytes to optimize their therapeutic benefit while attenuating the harmful effects of them.

## Overview of TBI and astrocyte

### Traumatic brain injury

Traumatic brain injury is a prevalent disease, with a global annual burden of approximately $US400 billion [2, 3]. According to statistics by the World Health Organization, TBI affiliated mortalities and disability will surpass that of many diseases as from the year 2020 [19]. However, there are currently no effective therapies for TBI [6, 7]. And the main form of clinical treatment is restricted to surgical interventions and supportive managements, including hyperbaric oxygen, task-oriented functional electrical stimulation, non-invasive brain stimulation, and behavioral therapy [6, 20]. One of the main challenges of treating TBI is the heterogeneity of its pathologic and pathogenic mechanisms. Consequently, an in-depth elucidation of the underlying pathophysiological mechanisms is required to provide new therapeutic targets.

### The pathophysiology of TBI

Traumatic brain injury is characterized by instant damage to mechanical force and delayed damage to the subsequent pathophysiological processes [21]. The mechanical force directly leads to neuronal or diffuse axonal damage and vascular disruption, followed by secondary injury mediated by extensive neuroinflammation, dysfunction of the BBB, oxidative stress, and apoptosis [22–26]. While the immediate primary injury is considered untreatable, the delayed secondary injury gives a window for intervention and has, therefore, attracted a lot of attention [27].

**Fig. 1:**
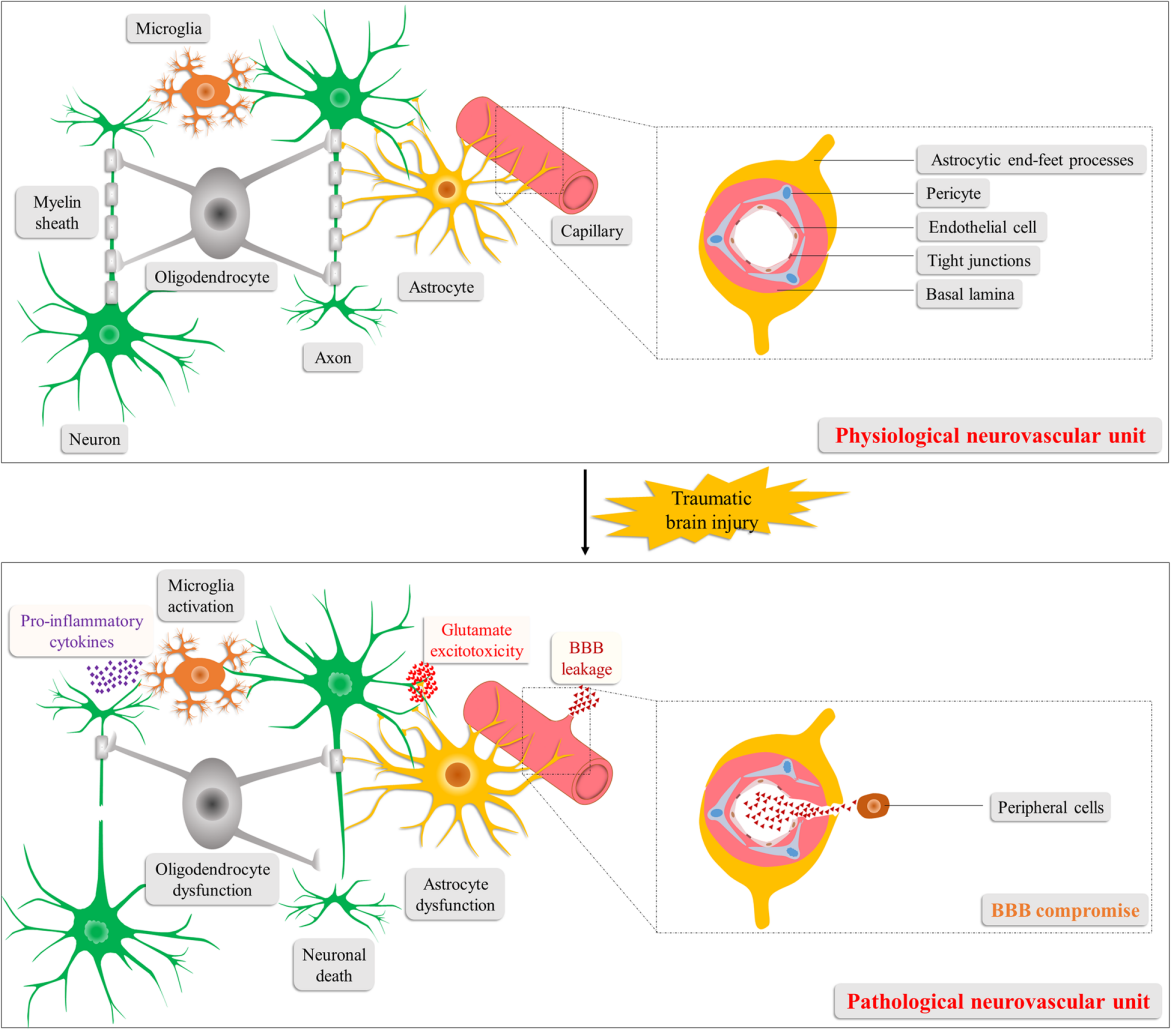
Schematic illustration of the neurovascular unit under normal physiological conditions and TBI pathological conditions. The neurovascular unit encompasses neurons, glial cells (astrocytes, oligodendrocytes and microglia), vascular cells (pericytes, endothelial cells and vascular smooth muscle cells) and the basal lamina matrix. Following TBI, disruption of the neurovascular unit arises from and further aggravates the pathophysiological processes of TBI, which include BBB compromise, neuronal death, neuroglial dysfunction, neuroinflammation, and metabolic disturbances

Following the initial injury, local environment changes and damaged cells release intracellular components, triggering the activation and recruitment of resident glial cells in the brain as well as the production of various cytokines, chemokines, and excitotoxins; then the peripheral immune cells are recruited into the brain with further release of signaling factors to induce a robust sterile immune reaction [28–30]. A broad range of literature data has reported the up-regulated expression of cytokines including interleukin (IL)-1β, tumor necrosis factor (TNF)-α, transforming growth factor-β (TGF-β), interferon γ (IFNγ), IL-6, IL-10 and IL-12 as well as the chemokines such as chemokine (C-C motif) ligand (CCL)2, CCL3, CCL4, chemokine (C-X-C motif) ligand (CXCL)1, CXCL2, CXXL4, CXCL8/IL-8 and CXCL10 in the early stages post-TBI, which boost the sterile inflammation [28, 31]. These lead to additional attraction of peripheral cells, continuous activation of resident glial cells, and aggravated neuronal damage [28, 32]. Disruption of the BBB integrity and the neurovascular unit (Fig. 1) can occur as a result of the initial injury or arise secondarily to the extensive neuroinflammation, astrocytic dysfunction, and metabolic disturbances. These damages result in vascular leakage, brain edema, cerebral hemorrhage, and hypoxia [27, 29, 33–35]. Neuronal apoptosis also significantly contributes to secondary injury [36, 37]. In addition to apoptosis, necroptosis, a recently identified programmed cell death bearing resemblance to both apoptosis and necrosis, has also been demonstrated to play an indispensable role in secondary neuronal cell death and neuroinflammation post-TBI [38, 39]. Mechanically, upon pathogenic stimuli following TBI, TNF-α-induced receptor-interacting protein 1 activation contributes to the formation of the so-called necrosome, a complex necessary for necroptosis [40, 41]. And after necroptosis, inflammatory factors released from damaged cells flow into the extracellular space, boosting the neuroinflammation [41–43]. All these primary or secondary pathologic mechanisms contribute to cell death, tissue loss, structural and metabolic abnormalities, and an ultimate neurological dysfunction in the patients [15, 44]. And whether neural structure and function can be restored determines the final outcome of the TBI patients [36].

### Astrocyte reaction after TBI onset

**Fig. 2:**
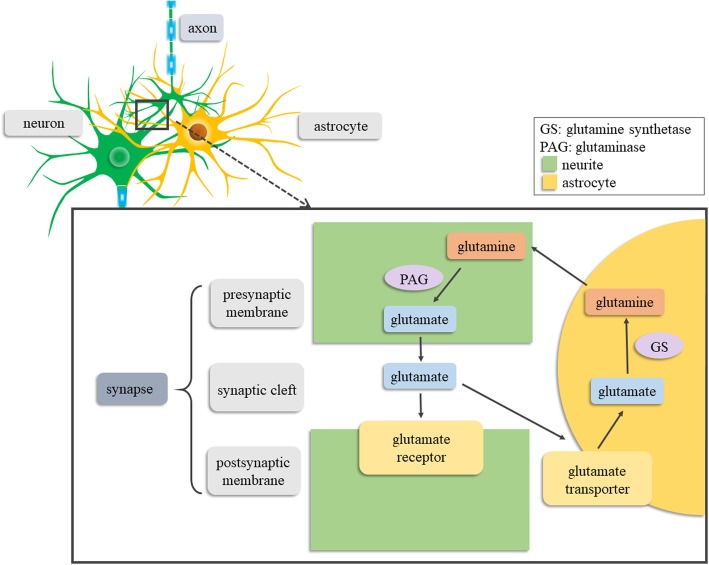
Schematic illustration of the glutamate-glutamine cycle in astrocytes. Astrocytes play a crucial role in the glutamate cycle of glutamate-glutamine. After the presynaptic membrane releases neurotransmitter glutamate, astrocytes can take in glutamate from the synaptic cleft through the glutamate receptor and synthesize glutamine with the catalysis of glutamine synthetase. And the glutamine can cross the cell membrane into the cytoplasm of presynaptic membrane and be deaminated by glutaminase to produce glutamate

Among brain resident glial cells such as astrocytes (astroglia), oligodendrocytes and microglia, astrocytes are the most abundant [45]. Astrocytes are characterized by the presence of glial fibrillary acidic protein (GFAP), a unique structural protein [45]. Under normal physiological conditions, astrocytes are involved in the homeostasis and blood flow control of the CNS [12]. Astrocytes structurally support neurons and separate the CNS from the meninges, blood vessels, and perivascular spaces by the creation of a functional barrier named glia limitans, which is formed via the interaction of astrocytic foot processes with the parenchymal basement membrane [46]. In addition, astrocytes provide functional support for neurons, including the recycling of the neurotransmitter glutamate, the most potent neurotoxin in the brain, via glutamate transporters (Fig. 2), the glutamate-glutamine shuttle system, and cystine–glutamate antiporter system [47–49]. Astrocytes play a role in the release of neurotrophic factors and gliotransmitters such as glutamate, ATP, γ-aminobutyrate (GABA), and D-serine [1, 15, 50]; the synthesis of glutamine, cholesterol, superoxide dismutases, glutathione, ascorbate and thrombospondin (TSP)-1 and 2 [9, 51, 52]. Astrocytes are also involved in the regulation of energy metabolism by the conversion of glucose into lactate [53–55] and the regulation of neuronal activation and water homeostasis through extracellular ion concentrations [56–59]. Given the multifunctional roles of astrocytes in the CNS, they can affect neuronal activity, modulate plasticity, and participate in CNS regeneration after brain injury [60–64].

Microglia are cells of myeloid origin and are considered “the CNS professional macrophages”, which express a large repertoire of pattern-recognition receptors and are often the first cells responding to any inflammatory events [29, 65]. Importantly, more and more lines of evidence suggests that astrocytes also express a series of receptors related to inflammatory and immune processes, including Toll-like receptors, purinergic receptors, mannose receptors, scavenger receptors, nucleotide-binding oligomerization domain proteins, double-stranded RNA dependent protein kinase, and components of the complement system, through which they sense a wide range of endogenous and exogenous signals and respond dynamically to sterile injuries and infectious non-self [29, 65–67]. Therefore, danger signals post-TBI can trigger inflammasomes and innate immune response via their interaction with the receptors on the innate immune neuroglia. Mechanically, when the local biochemical environment changes following the onset of TBI, danger signals induce the structural and functional alterations of astrocytes, including hypertrophy and increased expression of the intermediate filaments (nestin, vimentin, and GFAP), resulting in astrocyte activation (reactive astrogliosis) [15, 68]. Other cells such as brain-resident microglia are also activated [31]. Both astrocytes and microglia react within 24 hours and peak around day 3-7, however, microglia rapidly decline to control levels approximately 21 days after the lesion while astrocytes exhibit a long-lasting proliferative response, at least, 28 days after TBI [69–71]. The activation and proliferation of glial cells, in turn, have utility in releasing signaling factors and triggering a robust sterile immune reaction that consists of brain-resident as well as peripherally recruited inflammatory cells. This reaction is initiated to exert neuroprotective effects and promote wound healing, but may become maladaptive over time [29, 72].

As the inflammatory response progresses, local astroglial progenitors around the injured tissue form the glial scar that isolates the damaged area, contains the spread of inflammatory cells, provides a favorable environment for surviving neurons, and maintains the integrity of the BBB [46, 68, 73–75]. Nonetheless, the glial scar is considered the main hindrance to axonal regeneration and recovery of neuronal connectivity [76, 77]. This shows one of the Janus-like effects of astrocytes. Controversy also remains as to whether reactive astrogliosis is beneficial for the maintenance of BBB integrity after TBI [21, 78], since astrocytes can largely affect BBB integrity and water homeostasis [79] as detailed below: (1) the BBB is sheathed by perivascular astrocyte foot processes [80]; (2) the glymphatic system is formed by astrocytes [81]; (3) the perivascular aquaporin-4 (AQP4) is densely and exclusively expressed in astrocyte end-feet [82]; (4) the permeability of the BBB can be affected by astrocyte-derived factors [78]; and (5) the concentration of extracellular ions is controlled by astrocytes [9]. These “irrational” phenomena can be caused by an overreaction and dysfunction of reactive astrocytes after brain injury, or due to the release of neurodeleterious molecules [78]. Astrocytes, therefore, hold both neuroprotective and neurodeleterious effects following TBI, making it a double-edged sword for neurorestoration [83–85]. This also indicates that we cannot simply suppress or promote reactive astrogliosis, but should selectively stimulate the beneficial effects and ameliorate the deleterious ones in the astrocyte-targeting therapy [78].

## Dual roles of astrocytes in plasticity and reconstruction after TBI

As previously mentioned, all the primary and secondary pathologic mechanisms underlying TBI contribute to cell death, tissue loss, structural and metabolic abnormality, and ultimately lead to neurological dysfunction of TBI patients [15, 44]. And the ability to restore neural structure and function determine the outcome of the patients [36, 86]. Thus, promoting the astrocytes/astrogliosis-induced neuroprotective effects/molecules or attenuating the neurodeleterious ones in terms of neuronal regeneration and tissue reconstruction may represent a promising therapeutic target for TBI. Below, we will describe the astrocytes and a range of astrocyte-derived molecules, as well as their roles in neurogenesis, synaptogenesis, angiogenesis, blood-brain barrier repair, and glial scar formation after neurotrauma.

### Neurogenesis

Emerging evidence has indicated that astrocytes play a vital role in neurogenesis, which is attributed to the regulation of the microenvironment of neurogenic niche [87, 88].

#### The neurogenesis-promoting effects of astrocytes

Some studies suggested a beneficial effect of astrocytes in neurogenesis, both through the instruction of neuronal fate commitment and the promotion of proliferation of adult neural stem cells [88]. In addition, the neurogenesis-promoting effect of astrocytes has regional characteristics: hippocampal-derived astrocytes retain this potential, whereas astrocytes from the adult spinal cord do not [88]. Currently, some potential mechanisms concerning astrocytes-induced neurogenesis have been proposed. Astrocytes produce the neurotrophic and mitogenic protein S100β *in vivo.* Intraventricularly administration of S100β enhances neurogenesis within the hippocampus and improves cognitive function recovery following TBI. These improvements are mediated by the facilitation of neuronal differentiation, proliferation, and survival of hippocampal progenitor cells [89, 90]. Heme oxygenase induced by astrocytes after TBI catalyzes heme to carbon monoxide (CO), ferrous iron, and biliverdin. Notably, low concentrations (lower than 250 ppm possibly) of CO exert promotive effects on neurogenesis, as well as synaptic plasticity and angiogenesis [91]. Moreover, previous studies reported that mature astrocytes might regress to an immature phenotype and show stem cell characteristics [92].

Besides stimulating stem cell genesis, astrocytes also contribute to the prolonged survival of newborn neurons [93]. Neurotrophic factors secreted by astrocytes are closely involved in neuronal support and survival, and intraperitoneal administration of a formulation composed of co-ultramicronized palmitoylethanolamide and luteolin was found to promote this process [94, 95]. Additionally, pituitary adenylate cyclase-activating peptide expressed by astrocytes plays a significant role in the support and survival of new neurons post-TBI [93]. Both the enhanced neurogenesis and long-lasting survival of newborn neurons result in a better neurological recovery.

#### The neurogenesis-suppressing effects of astrocytes

However, under certain pathological conditions, such as severe TBI with devastating excitotoxicity and inflammatory response, the microenvironment of neurogenic niche may lose its homeostasis [21, 96]. Correspondingly, some studies proposed that knockout/knockdown of molecules produced by astrocytes or suppression of astrocyte-related signaling enhances neurogenesis. Mice devoid of GFAP and vimentin are found to be developmentally normal with increased hippocampal neurogenesis and axonal regeneration post-TBI, despite that GFAP is essential for astrocyte activation and acute cellular stress handling [97–100]. This disparity may be due to the mechanism that differentiation of uncommitted neural progenitor cells is skewed towards neuronal lineage under the null of *GFAP* gene condition, and inhibition of Sirt1 expression may strengthen this inclination [101]. The effects and mechanisms of several GFAP suppressors have also been evaluated in experimental TBI [45].

Garber *et al.* revealed that astrocytes impaired neuronal progenitor cell homeostasis via the up-regulated expression of IL-1, thus hindering hippocampal neurogenesis in West Nile virus neuroinvasive disease, which could be reversed by IL-1R1 antagonist [83]. Upregulated IL-1β is also found to aggravate excitotoxicity and seizures post-TBI, although the latter can develop independently from the neurotoxic effects [102, 103]. Interestingly, Barkho *et al.* suggested that IL-1β and IL-6 could promote neuronal differentiation of neural stem/progenitor cells at relatively low concentrations and thus they proposed a concentration-depending effect of astrocyte-derived pro-inflammatory cytokines. They also indicated that three other astrocyte-derived molecules: insulin-like growth factor (IGF) binding protein 6 and decorin, which inhibit IGF and TGF-β respectively, and opioid receptor agonist enkephalin, could inhibit neurogenesis [104].

### Synaptogenesis

Astrocytes also play a crucial role in synaptic plasticity, remodeling, and regeneration post-TBI [105, 106]. As mentioned earlier, astrocytes are involved in the biochemical synthesis, metabolism, and secretion of many molecules. Some of these molecules, such as TSP-1 and TSP-2, promote synaptogenesis, while molecules, including trophic factors and cholesterol, preserve synapse maturation and maintenance [106–108]. Reversely, these mechanisms (and others) are also potentially critical for eliciting pathological responses during and after TBI [87, 109].

#### The synaptogenesis-promoting effects of astrocytes

Several studies have reported the beneficial role of astrocytes in synaptogenesis, which is reflected in its involvement in synaptic formation, metabolic support, and neurotransmitter release [9, 110]. For instance, astrocytes regulate the expression and localization of agrin, one of matrix metalloproteinase (MMP)-3 substrates, which induces reactive synaptogenesis and neurological recovery [111]. And astrocytes support ovarian steroids estradiol-enhanced neurite outgrowth, although this can be antagonized by activated microglial-induced progesterone [112]. Remarkably, astrocytic signal transducer and activator of transcription-3 (STAT3) is capable to regulate the process formation and re-expression of TSP-1 of perineuronal astrocytes [18]. Furthermore, STAT3 supports neuronal integrity and mediates anti-inflammatory reactions [18, 113, 114]. The augmentation of STAT3 discloses a neuroprotective effect, whereas the conditional ablation of STAT3 has the opposite effect [113, 114]. Nevertheless, Christopherson *et al.* demonstrated that TSP-induced excitatory synapses are postsynaptically silent, which owes to the lack of functional α-amino-3-hydroxy-5-methyl-4-isoxazole propionic acid (AMPA) receptors [115]. Similarly, Kucukdereli *et al.* demonstrated that hevin, another matricellular protein secreted by astrocytes, could induce the same type of synapse as TSP [116]. On the contrary, the homologous sequence protein, secreted protein acidic and rich in cysteine (SPARC) inhibits hevin-induced synapse formation [116–118]. Other astrocyte-derived molecules such as glypicans [119], TGF-β [120, 121], and brain-derived neurotrophic factor [122] can induce excitatory synapse formation, while γ-protocadherin can induce the formation of either excitatory or inhibitory synapse via a contact-dependent mechanism [123].

#### The synaptogenesis-suppressing effects of astrocytes

Following the breakdown of the BBB, an influx of serum elements, and the inflammatory cytokines, including IL-1, TGF-β trigger the formation of a glial scar to cope with injury. Nonetheless, the glial scar is considered the main hindrance to axonal regeneration and neuronal connectivity recovery, due to the production of growth-inhibitory components and the formation of physical and chemical barriers that hinder axon elongation [30, 76, 77, 124]. Among the inhibitory components, chondroitin sulfate proteoglycans (CSPGs), one of the ECM molecules produced by astrocytes, are of prime importance as they are predominantly responsible for the non-permissive characteristic of glial scar and have been extensively studied [125–128]. The major brain CSPGs include lecticans (neurocan, brevican, versican, and aggrecan), phosphacans, and transmembrane NG2; they surround and affect the perineuronal nets (PNNs), which are comprised of rich ECM and cell adhesion proteins and have been found to stabilize synapses [129, 130]. The class IIa/Leukocyte common antigen-related (LAR) family [131] and the NOGO receptors NgR1 and NgR3 [132] have been identified as CSPG receptors and convey subsequent axonal growth inhibition. However, heparan sulfate proteoglycans (HSPGs), another ligand for the LAR family receptors promotes axon extension [133]. This role of HSPGs may result from the switch of axonal endings between states of growth and inactivity via the oligomerization status of PTPσ (a member of the LAR family) [76]. Therefore, agents targeting these receptors or that mimic HSPG binding may mitigate the inhibitory environment of glial scar and augment neuronal regeneration, thus suggesting multiple candidates of therapeutic application for TBI [134, 135]. For instance, the hepatocyte growth factor, which exhibits pleiotropic functions in the CNS has been shown to suppress the expression of CSPGs after brain injury, as well as block the secretion of TGF-β1 and β2 and the subsequent induction of the glial scar [124]. Another ECM component, tenascin-C, was also shown to inhibit axon outgrowth and therefore represents a target for intervention [136, 137].

Matrix metalloproteinases cleave ECM and are involved in the modulation of synaptogenesis. However, the definite role of MMPs in neurological recovery post-TBI remains elusive, since it depends on where and when it is activated [138]. After severe TBI, astrocytes induce the expression of MMP-3 in a higher and more persistent pattern, resulting in maladaptive synaptogenesis and poor recovery of neural function, while MMP inhibitor FN-439 is shown to attenuate the activity of MMP-3 and then facilitate functional recovery [139]. Moreover, persistent expression of another MMP, *a d*istintegrin *a*nd *m*etalloproteinase-10 (ADAM-10), parallels the attenuation of the N-cadherin level, which is critical to synapse stability, and consequently contributing to reduced functional recovery; whereas inhibition of MMP shifts the expression of ADAM-10 and N-cadherin towards an adaptive pattern and facilitates the synapse formation [17].

### Synaptic plasticity

In addition to the number of synapses, synaptic plasticity is also necessary for learning and memory formation. Synaptic plasticity can be influenced by activation and localization of glutamate receptor, synaptic strength, intracellular calcium levels, neurotrophic factors, and cytokines following TBI [140–142]. Considering the involvement of astrocytes in the pathophysiological processes including supporting neuronal metabolism, secreting different molecules that induce the formation of excitatory synaptic structure and function, and releasing gliotransmitters that affect the balance of neural network as well as synaptic potentiation or depression, astrocyte may be a promising target for modulating synaptic plasticity [87]. Following TBI, the general role of astrocytes in synaptic plasticity again remains obscure. For instance, the sphingosine 1-phosphate (S1P) receptor 1 antagonist siponimod preserves neural plasticity via attenuating activation of astrocytes, microglia, and other inflammatory cells [143]. On the contrary, minocycline influences neuronal plasticity and improves neurological recovery by increasing the astrogliosis following experimental stroke [144].

#### The synaptic stability-promoting effects of astrocytes

The previously mentioned neurogenesis-promoting CO also facilitates synaptic plasticity [91]. Besides, the synaptogenic factor TSP-1 can also suppress MMP-9-induced cleavage of extracellular matrix molecules and synaptic instability [145, 146]. AQP4, which is the main water channel of astrocytes and exclusively expressed on astrocytes, plays a critical role in synaptic plasticity and memory encoding [147]. Moreover, AQP4 is also highly correlated with the balance of water, the function of glymphatic pathway and the integrity of the BBB while the role of AQP4 may, however, depend on the stage of TBI progression [147, 148]. The study by Zhang *et al.* revealed that lack of AQP4 could lead to the accumulation and removal of excess water in the brain during acute and late stages of TBI, respectively [149], making AQP4-targeting therapy a great challenge.

Although the glial scar is regarded as the main impediment to axonal regeneration and neuronal connectivity recovery, it initially acts as a barrier isolating the damaged area, containing the spread of inflammatory cells, providing a favorable environment for surviving neurons and maintaining the BBB [30, 76, 150, 151]. Moreover, despite the detrimental roles mentioned above, CSPGs may help restrict inflammation by shifting monocytes towards resolving phenotype and enhancing the expression of anti-inflammatory cytokines, such as IL-10, as well as help stabilize the ionic microenvironment by limiting diffusion of cations, such as potassium, calcium, and sodium [152, 153]. Furthermore, CSPGs [154, 155] and TNF-α [156–158] have been demonstrated to alter the level or mobility of AMPA receptors in a beneficial manner, which are critical in synaptic plasticity. Consistent with these findings, several studies have reported that most of the ECM molecules produced by astrocytes elicit both restrictive and permissive effects on axonal sprouting post-lesion [79]. Indeed, studies demonstrated that ablation of astrogliosis in transgenic mice disrupted scar formation, which in turn exacerbated the spread and persistence of inflammation response, vasogenic edema, neuronal loss, demyelination, and functional recovery [159–162]. Furthermore, blocking scar formation in STAT3 deletion mice has similar effects of inducing extensive lesions and increasing neuronal loss and locomotor deficits after CNS injury, while enhancing scar formation in protein suppressor of cytokine signaling 3 deletion mice has the opposite effects [113, 114]. These findings strongly suggest that astrogliosis and glial scar formation may be neuroprotective against brain damage under particular circumstances, highlighting a dichotomous role again.

#### The synaptic stability-suppressing effects of astrocytes

Astrocytes play a crucial role in regulating excitatory chemical transmission via glutamate transporters (Fig. 2), glutamate-glutamine shuttle system, and cystine–glutamate antiporter system. However, the impairment of astrocytic glutamate uptake and GABA release lead to glutamate excitotoxicity as well as ion and water imbalance post-TBI [1, 9]. Glutamate is the primary excitatory neurotransmitter and the most potent neurotoxin once concentrated in the extracellular space of CNS. Notably, the homeostasis of glutamate is closely associated with synaptic plasticity [47–49]. Ephrin-A3, a member of the ephrin family, is expressed in astrocytes and is involved in the regulation of glial glutamate transporters. Ephrin-A3 is required for maintenance of long-term potentiation via its interaction with the A-type Eph receptor, namely EphA4, and thus influences synaptic plasticity. Once Ephrin-A3 is over-expressed following TBI, it decreases glutamate transporters and increases glutamate excitotoxicity, hence prolonging neuronal depolarization and focal dendritic swelling [163–165]. Therefore, inhibition of Ephrin-A3 represents a potential therapeutic strategy. Besides, the glutamate receptor antagonist MK-801 has also been shown to enhance synaptic integrity and improve cognitive outcomes [138, 139].

Traumatic brain injury constitutes one of the most common causes of acquired epilepsy [166]. Epileptogenesis can be induced by several pathological processes, including glial scar, ECM remodeling, axonal plasticity alteration, excitation/inhibition imbalance, cell death, and neuronal heterotopia [167]. Once the structural integrity of PNNs is compromised by astrocyte-derived ECM molecules, dysfunctional PNNs around the fast-spiking inhibitory interneurons might underlie excitation/inhibition imbalance and lead to the development of post-traumatic epilepsies [168]. The involvement of hyperphysiologic TNF-α in post-traumatic epileptogenesis has also been revealed [169, 170]. In addition to its influences on glutamatergic transmission and synaptic plasticity, TNF-α also has an important role in the initial activation of microglia and astrocytes and the disruption of the BBB; and the biologic TNF antagonist etanercept was shown to improve the outcomes of experimental TBI [171]. Furthermore, astrogliotic upregulation of enzyme adenosine kinase also contributes to epileptogenesis [172]. Notably, TBI is also an important risk factor for the development of many neurodegenerative diseases such as Alzheimer’s disease, chronic traumatic encephalopathy, amyotrophic lateral sclerosis, and etcetera; the deposition and accumulation of amyloid-beta and tau are considered as part of the pathological mechanisms [173–176].

### BBB repair

Although TBI-induced astrogliosis and glial scar seem to promote the BBB repair [30], astrocytic dysfunction is one of the main pathological mechanisms giving rise to the BBB disruption post-TBI [27, 29, 33]. The dual roles of reactive astrogliosis owe to the distinct functions of various astrocyte-derived molecules in BBB integrity [78] (**Table****1**). Furthermore, these astrocyte-derived factors also regulate cell adhesion molecules on the endothelial cells, thereby controlling the leukocyte infiltration influx to the CNS, and participate in one or more pathophysiological processes including angiogenesis, neurogenesis, and neuroplasticity [78].

**Table 1:** Dual roles of astrocyte-derived factors in the BBB integrity after TBI

Astrocyte-derived factors	Characters	Receptors	Role in BBB post-TBI	Mechanisms	Related agents	Other functions	References
ANG-1	Glycoprotein	Tie-2	Protect	Promote endothelial cells, vascular remodeling, and stability; increase TJ-related proteins	Exogenous ANG-1 or ANG-1 mimetic peptides	Promote angiogenesis; suppress VEGF-induce expression of cell adhesion molecules and leukocyte infiltration	179-181
SHH	Glycoprotein	Patched-1	Protect	Attenuate endothelial cells apoptosis; increase TJ-related proteins	Exogenous SHH	Promote angiogenesis; promote normal pattern formation and cellular differentiation in the developing CNS; suppress cell adhesion molecules expression and leukocyte infiltration	182-185
GDNF	Neurotrophic factor	GDNF receptor α-1 and -2	Protect	Increase TJ-related proteins	Exogenous GDNF	Promote the normal postnatal development of BBB, neuronal survival and angiogenesis; axon guidance and synapse formation; control endothelial functions;	186-188
RA	Active metabolite synthesized from retinol by retinaldehyde dehydrogenase	Nuclear RA receptors	Protect	Increase TJ-related proteins and vascular endothelial cadherin	Exogenous RA	Promote growth and development in the CNS; regulate synaptic plasticity; suppress the expression of cell adhesion molecules	189-191
IGF-1	A member of insulin gene family	IGF-1 receptors	Protect	Attenuate endothelial cells apoptosis	Exogenous IGF-1	Promote neurogenesis; reduce cell death; support injury repair; regulate synaptic neuroplasticity	78, 192, 193
APOE	A member of the apolipoprotein family	\	Protect*	Suppress the activity of MMP-9; increase TJ-related proteins	APOE-mimetic peptide COG1410	Support lipid transport and injury repair	194-203
VEGF	An angiogenetic factor	VEGFR-1 and VEGFR-2	Damage	Decrease TJ-related proteins	SU5416 (VEGFR-2 inhibitor); cavtratin (a selective inhibitor of VEGF-A)	Promote endothelial proliferation and differentiation for angiogenesis; induce cell adhesion molecules expression and leukocyte infiltration	204-207
MMP	Zinc-endopeptidases	\	Damage	Enhance endothelial cell apoptosis; degrade TJ-related proteins and ECM molecules	Ro32–3555 (a broad spectrum MMP inhibitor)	Promote angiogenesis; regulate expression of cell adhesion molecules and subsequent leukocyte infiltration	208, 213, 214
NO	A potent vasodilator synthesized from L-arginine by NO synthase	\	Damage	Enhance MMPs activation; decrease TJ-related proteins; induce apoptosis through cGMP monophosphate-independent pathways, suppress apoptosis through cGMP pathway	Nomega-Nitro-L-arginine methyl ester (a non-specific NOS inhibitor)	Regulate blood flow for neuronal activity; exacerbate inflammatory reaction	78, 215, 216
Glutamate	A major excitatory transmitter and	NMDA receptor and the AMPA receptor	Damage	Induce excessive vascular permeability via activation of NMDA receptors; decrease TJ-related proteins	MK-801 (non-competitive NMDA receptor antagonist); CGS-19755 (competitive NMDA receptor antagonist); NBQX, DNQX (competitive AMPA receptor antagonists)**	Regulate synaptic plasticity and formation; induce vasodilatation; regulate neuronal survival	208-210
ETs	Potent endogenous vasoconstrictors	Endothelin receptor type A/B	Damage	Exacerbate BBB inflammation; enhance MMPs activation; degrade TJ-related proteins	S-0139 (selective ETA receptor antagonist); BQ788 (selective ETB receptor antagonist)	Induce expression of cell adhension molecules; regulate endothelial function	21, 211, 212
S1P	A biologically active lipid	S1PR 1-5	Dual	Regulate VEGF activation and TJ-related proteins	Siponimod, fingolimod, TASP0277308 (antagonists of S1PR 1); artesunate, isoflurane (agonists of S1PR 1)^✝^	Regulate synaptic plasticity	143, 222-226

*APOE exerts its regulation of BBB integrity in an isoform-dependent manner, APOE4 activates the activity of MMP-9 and accelerates the BBB permeability^195, 196^

**Some studies suggested that blockade of AMPA receptor did not promote glutamate-mediated BBB breakdown^210^

^✝^Both the antagonists which suppress the activation of S1PR 1 and agonists which activate S1PR 1 have been demonstrated to preserve the BBB integrity^143, 222-226^

*Abbreviations*: *ANG-1* angiopoietin-1, *TJ* tight junction, *VEGF* vascular endothelial growth factor, *BBB* blood-brain barrier, *TBI* traumatic brain injury, *SHH* sonic hedgehog, *GDNF* glial-derived neurotrophic factor, *RA* retinoic acid, *IGF-1* insulin-like growth factor-1, *APOE* apolipoprotein E, *MMP* matrix metalloproteinases, *ECM* extracellular matrix, *NO* nitric oxide, *cGMP* cyclic guanosine, *NMDA* N-methyl-D-aspartate, *ETs* endothelins, *S1P* sphingosine 1-phosphate, *AMPA* α-amino-3-hydroxy-5-methyl-4-isoxazolepropionic acid, *S1PR* S1P receptor

#### The BBB integrity-promoting effects of astrocytes

The integrity of the BBB is determined by the endothelial tight junctions and the basal lamina. While endothelial tight junctions are formed by proteins such as claudin, occludin and zonula occluden (ZO), the basal lamina forms the basement membrane of ECM and includes laminin, collagen, and fibronectin [177, 178]. Astrocyte-derived factors including angiopoietin-1 (ANG-1) [179–181], sonic hedgehog (SHH) [182–185], glial-derived neurotrophic factor (GDNF) [186–188], retinoic acid (RA) [189–191], and IGF-1 [192, 193] have been demonstrated to promote recovery of the BBB by protecting endothelial cells and/or enhancing tight junction reassembly, via signaling mediated by their receptors, tie-2, patched-1, GDNF receptor alpha-1 and alpha-2, nuclear RA receptor, and IGF-1 receptor, respectively [78, 79] (Table. 1). Besides, the astrocyte-secreted apolipoprotein E (APOE) isoforms APOE2, APOE3, and APOE4, are also closely involved in the regulation of BBB integrity [194]. Notably, APOE exerts its regulation in an isoform-dependent manner [195]. Despite that APOE3 protects against BBB disruption via the suppression of a cyclophilin A (CypA)-nuclear factor-κB (NFκB)-MMP-9 pathway, APOE4 activates the pathway and results in neuronal dysfunction and degeneration [196]. Overall, APOE tends to maintain BBB integrity and promote neurological recovery. While APOE-deficiency provokes BBB dysfunction, exogenously administered APOE or its mimetic peptides preserve BBB integrity in experimental studies [197–203].

#### The BBB integrity-suppressing effects of astrocytes

Despite that some astrocyte-derived factors maintain the BBB function, some astrocyte-derived factors damage the BBB by inducing endothelial cell apoptosis or decreasing the expression of endothelial tight junction-related proteins, which include vascular endothelial growth factor (VEGF) [204–207], glutamate [208–210], endothelins (ETs) [21, 211, 212], MMP [208, 213, 214], and nitric oxide (NO) [215, 216] (Table 1). As zinc-endopeptidases, MMPs can directly degrade endothelial tight junction-related proteins and ECM molecules, which promotes angiogenesis whereas simultaneously increases BBB permeability [78, 217, 218]. And it is through the signaling pathway activating or suppressing MMPs that many other factors such as APOE, NO, and ETs get to affect the BBB integrity [201, 212, 215]. Although both NO and glutamate can decrease endothelial tight junction-related proteins, NO may have inconsistent effects on apoptosis through different pathways [219]. Furthermore, glutamate also exacerbates vascular permeability via the activation of glutamate receptors [220], and cytokines such as TNF-α are strictly related to BBB disruption [171, 211, 221].

The study by Prager *et al.* indicates that S1P binds to and activates five G protein-coupled receptors. Among these receptors, S1P receptor 1 (S1PR1) primarily preserves BBB integrity while the S1P receptor 2 damages integrity [222] and correspondingly, agents activating S1PR1 such as artesunate and isoflurane have been demonstrated to preserve the BBB integrity [223, 224]. However, several antagonists which suppress the activation of S1PR1 have also been found to preserve the BBB integrity [143, 222]. Remarkably, the S1PR1 antagonist fingolimod (FTY720) can also possibly induce S1P1 activation [225]. These observations suggest that S1PR1 plays a dual role in BBB permeability, depending on the ligand, which is in line with the assumption proposed by Schuhmann *et al.* [226].

## Usage of astrocyte and astrocyte-derived molecules as therapeutic targets

**Fig. 3:**
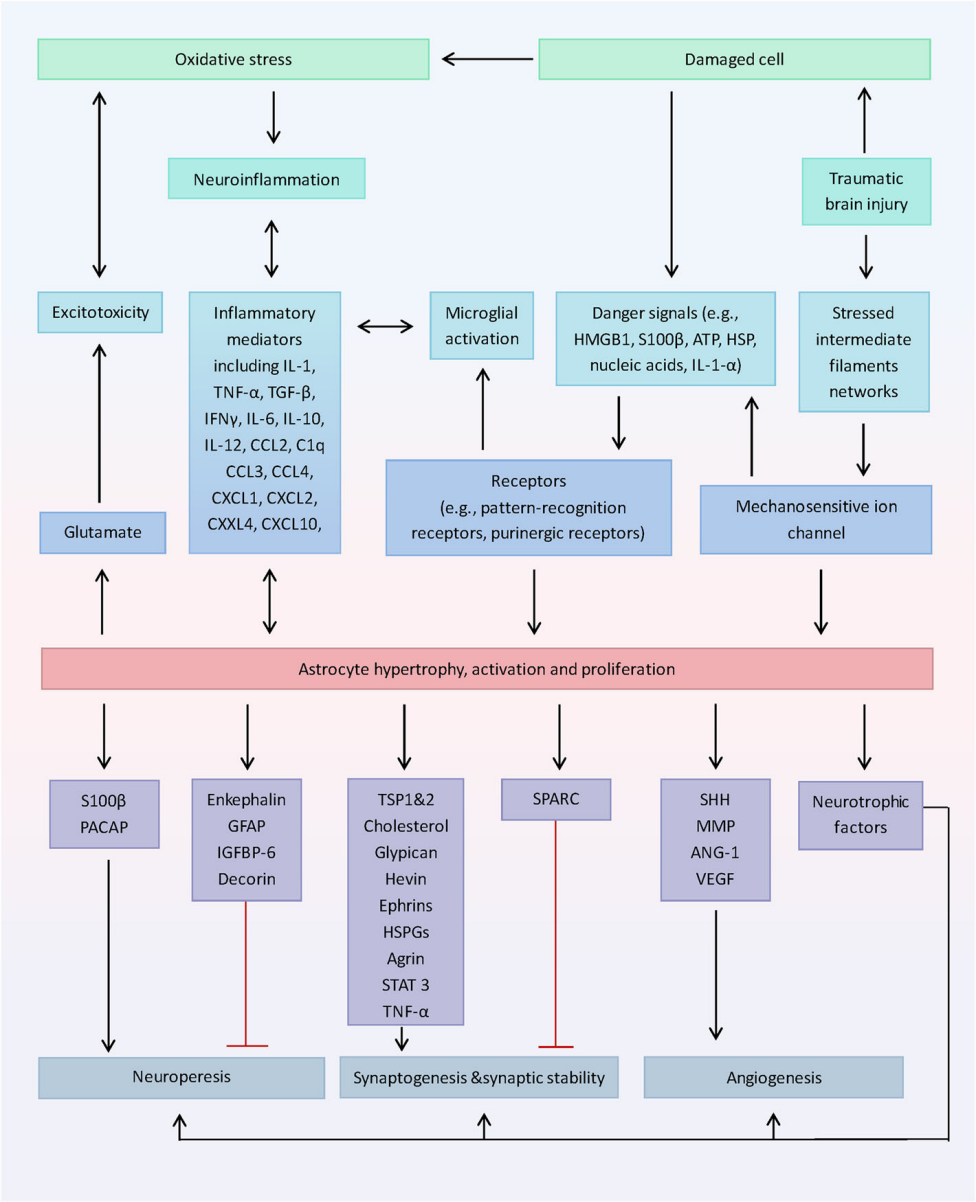
Potential therapeutic targets regarding astrocyte-derived molecules following TBI. Following TBI, damaged cells release danger signals. And stressed intermediate filaments networks within astrocytes activate ion influx through the mechanosensitive ion channel, resulting in the further release of danger signals. These signals serve to activate neuroglia and induce a robust sterile immune reaction and other secondary TBI pathogenesis. Reactive astrocytes secrete a wide range of factors that affect neurogenesis, synaptogenesis and synaptic stability, and angiogenesis, which may represent the therapeutic targets. Modulating the maladaptive microenvironment caused by neuroinflammation, excitotoxicity and oxidative stress post-TBI is also a considerable therapeutic strategy. ANG-1, angiopoietin-1; CCL, chemokine (C-C motif) ligand; CXCL, chemokine (C-X-C motif) ligand; GFAP, glial fibrillary acidic protein; HMGB1, high mobility group protein B1; HSP, heat shock proteins; HSPGs, heparan sulfate proteoglycans; IFN, interferon; IGFBP-6, insulin-like growth factor binding protein 6; IL, interleukin; MMP, matrix metalloprotein; PACAP, pituitary adenylate cyclase-activating peptide; SHH, sonic hedgehog; SPARC, secreted protein acidic and rich in cysteine; STAT3, signal transducer and activator of transcription-3; TBI, traumatic brain injury; TGF-β, transforming growth factor-β; TNF, tumor necrosis factor; TSP, thrombospondin; VEGF, vascular endothelial growth factor

As a result, all of the described neuroprotective and neurodeleterious molecules, as well as their upstream and downstream factors, represent potential therapeutic targets (Fig. 3 and Table 1). However, both astrocytes and astrocyte-derived molecules can only act as targets for particular subtypes, specific damage regions, and certain stages of TBI. Therefore, therapeutic strategies must focus on the enhancement of neuroprotective effects and blockage of the neurodeleterious effects of the different factors under specific conditions.

Besides targeting astrocyte-derived molecules, stimulating the function of astrocyte-related receptors is also promising for the restoration of neuronal plasticity and reconstruction. Some astrocyte-derived molecules such as S1P and ETs also act as ligands of astrocytic receptors, and the probable therapeutic drugs are shown in the Table 1. Other receptors such as Toll-like receptors [127], purinergic receptor [227], glutamate receptor [228], hormone receptor [10, 229], and cannabinoid receptor [230] have also attracted widespread attention. Although we previously mentioned that MK-801, one of the glutamate receptor antagonists, had been shown to enhance synaptic integrity and improve cognitive outcome in the experimental study; but regrettably, clinical trials concerning the glutamate receptor antagonists have been widely carried out but failed to provide a statistically significant benefit for TBI patients [231]. According to Ikonomidou *et al.,* the failure could be attributed to the attenuation of synaptic transmission, which impedes neuronal survival [228].

Modulating the maladaptive microenvironment post-TBI is also a considerable therapeutic strategy [140–142]. Relevantly, agents for reducing the glutamate excitotoxicity by enhancing glutamate transporters such as parawexin 1 and certain β-lactam antibiotics could be of therapeutic benefit [232, 233]. Other potential therapeutic mediators include agents for the restoration of ionic and water balance by targeting Na^+^/H^+^ transporters, Na^+^/K^+^/2Cl^−^ cotransporters, or Na^+^/Ca2^+^ exchangers such as fluorenyl drugs [234, 235] and agents that promote neuronal survival and function such as recombinant neurotrophins or peptidomimetics [9]. Agents that alter the lesion environment by modulating inflammatory responses such as minocycline and etanercept have also been proposed as potential candidates for neuroprotection [144, 171].

We have previously reviewed the advance of stem cell treatment for TBI, which has not reached a general success in clinic application [86]. Given the vital roles of astrocyte-secreted factors in the neurogenesis and neural differentiation, a combination of stem cell treatment and astrocytic functions may present a novel therapeutic strategy. Besides, non-coding RNAs also hold therapeutic potential as astrocytes express various non-coding RNAs, which in turn control astrocytic functions [236–238]. And hypertonic saline has been found to elicit neuroprotection by regulating the expression of non-coding RNAs [239].

## Conclusion and perspectives

In this article, we describe for the first time the detailed dual roles of astrocytes in the field of neuronal plasticity and reconstruction including neurogenesis, synaptogenesis, angiogenesis, BBB repair, glial scar formation after TBI, and attempt to classify astrocyte-derived factors by neuroprotection and neurotoxicity to make the targeted therapy more relevant and meaningful. However, not only astrocytes have a dual role, but some factors derived from astrocytes also have double-sided properties, which may due to the distinct microenvironment and molecular mechanisms underlying the different subtypes, different damage zone, and different stages of neurotrauma. For example, mild TBI and severe TBI will induce different physiological and pathological mechanisms as well as different astrocytic reaction; hippocampus-derived astrocytes and spinal cord-derived astrocytes boost different effects on neurogenesis; the acute and the late stages post-TBI elicit different roles of AQP4. Therefore, simply suppressing or promoting reactive astrogliosis does not have a satisfying curative effect, whereas selectively stimulating the beneficial astrocyte-derived molecules while attenuating the deleterious ones based on the spatiotemporal-environment represents a promising astrocyte-targeting therapeutic strategy. As far, there are a number of related animal experiments that provide some novel therapeutic targets for the pharmacotherapy of TBI, but related clinical trials are rare and the existing ones have failed to show promise for long-term prognosis. Future research should focus more strictly on distinguishing the various functions of astrocyte-derived molecules in a clear subtype, region, and stage of TBI. In addition, more clinical trials concerning astrocyte-targeting therapy are warranted.

## Data Availability

Not applicable.

## Abbreviations

ADAM-10: a distintegrin and metalloproteinase-10
AMPA: α-amino-3-hydroxy-5-methyl-4-isoxazolepropionic acid
ANG-1: Angiopoietin-1
APOE: Apolipoprotein E
AQP4: Aquaporin-4
BBB: Blood-brain barrier
CCL: Chemokine (C-C motif) ligand
cGMP: Cyclic guanosine
CNS: Central nervous system
CO: Carbon monoxide
CSPGs: Chondroitin sulfate proteoglycans
CXCL: Chemokine (C-X-C motif) ligand
CypA: Cyclophilin A
ECM: Extracellular matrix
ETs: Endothelins
GDNF: Glial-derived neurotrophic factor
GFAP: Glial fibrillary acidic protein
HSPGs: Heparan sulfate proteoglycans
IFN: Interferon
IGF-1: Insulin-like growth factor-1
IL: Interleukin
MMP: Matrix metalloprotein
NFκB: Nuclear factor-κB
NMDA: N-methyl-D-aspartate
NO: Nitric oxide
PNNs: Perineuronal nets
RA: Retinoic acid
S1P: Sphingosine 1-phosphate
S1PR: S1P receptor
SHH: Sonic hedgehog
SPARC: Secreted protein acidic and rich in cysteine
STAT3: Signal transducer and activator of transcription-3
TBI: Traumatic brain injury
TGF-β: Transforming growth factor-β
TJ: Tight junction
TNF: Tumor necrosis factor
TSP: Thrombospondin
VEGF: Vascular endothelial growth factor
ZO: Zonula occluden
